# A Long Way to Go Understanding the Role of Chaplaincy? A Critical Reflection on the Findings of the Survey Examining Chaplaincy Responses to Covid-19

**DOI:** 10.1177/1542305021992002

**Published:** 2021-03-17

**Authors:** Megan Best, Geila Rajaee, Anne Vandenhoeck

**Affiliations:** The University of Sydney, Sydney, Australia; University of Michigan, Ann Arbor, USA; KU Leuven, Leuven, Belgium

**Keywords:** Critical reflection survey, generalist versus specialist spiritual care, chaplains' role, telechaplaincy

## Abstract

This contribution reflects on some of the most prominent findings in the survey on the chaplaincy response to the COVID-19 pandemic. The finding that chaplain respondents had difficulty understanding their own role prior to the first wave is of concern. If chaplains cannot articulate their own role, it is not surprising that those around them are also unclear. Chaplains are not the only ones to blame for the confusion around their role though.

This special issue of the Journal of Pastoral Care and Counseling contains articles reporting on the chaplaincy response to the COVID-19 (novel severe acute respiratory syndrome coronavirus 2) pandemic. The pandemic has had immense global impact (World Health Organization, 2020) creating widespread anxiety and fear for many people. In some ways, it is exactly the situation where one might expect a chaplain to feel completely at ease, armed with the required skills to provide comfort in times of great spiritual need (Best et al., 2020). What is striking about this research is the breadth of reported experiences for chaplaincy services in the first wave of the pandemic, from being made redundant to receiving institutional support for increased visibility.

Such an occurrence immediately suggests a range in understanding of the role of chaplaincy. The finding that chaplain respondents, as a whole, had difficulty understanding their own role prior to the first wave of the pandemic is of concern. If chaplains cannot articulate their own role, it is not surprising that those around them are also unclear. There are many reasons why chaplains may feel unsure of their place in a healthcare setting (Best et al., 2020). Best and colleagues found that chaplains were unclear on the evidence for patient outcomes in response to spiritual care. Continued work on assessing outcomes as well as increasing the professionalisation of chaplaincy will be important in clarifying the role in the healthcare context.

In those countries where accreditation of chaplains remains absent or optional, there will be continuing challenges in persuading healthcare leadership that chaplains should be included in executive committees (and thus decision making), such as those developed to manage the COVID-19 crisis. Professional chaplaincy organizations are responsible for promoting and advocating for an agreed code of practice for chaplaincy care. The critique that chaplains, unlike other healthcare professionals, are not subject to a shared code of conduct across healthcare specialities and institutions highlights the need for better advocacy efforts. The current state of affairs may impede the ability of chaplains to engage with healthcare leadership and make it more difficult to be ‘at the table’ when important decisions are being made about patient care during times of crisis or even in day-to-day management. Membership in professional organizations is needed for advocacy to be successful, as well as for members to receive appropriate support in good times and bad. It is to be hoped that professional organizations will engage in greater advocacy and education regarding national codes of practice for chaplains within healthcare organizations. Additionally, educational programs for chaplains should include equipping them to advocate for their roles as spiritual care specialists.

Chaplains are not the only ones to blame for the confusion around their role. As chaplains are providing spiritual care to patients/residents, family members and staff on a daily basis, healthcare management and other key healthcare professionals remain oblivious to understanding chaplaincy as a speciality, providing a contextualised, multi-layered contribution to health care (Damen et al., 2019). There are widespread ongoing misconceptions that chaplains *exclusively* provide religious support that is easily replaced by the religious support from representatives of local faith communities. It seems that persistent memories of older, more ‘traditional’ models of care continue to define the perception of chaplains and chaplaincy. This effectively limits the practice and development of contemporary and emerging models of chaplaincy care. More research is needed to discover why these images remain persistent and how it is possible that despite modern chaplaincy practice and a growing number of research articles pointing towards the specialised contribution of chaplains, little seems to be changing with regard to this perception.

The lack of baseline data regarding who typically is involved in spiritual care makes it difficult to determine the significance of findings that generalist spiritual care was widely practiced (Snowden, Table 3). Recent promotion of interprofessional spiritual care models (Best et al., 2020; Puchalski et al., 2020) makes it unsurprising that interest in patients’ spiritual wellbeing would have been addressed by multidisciplinary staff members. It is encouraging to see that the space between generalist and specialist spiritual care was more fluid in a time of crisis, particularly if it occurred in areas where limited staff members had access to patients. This highlights the need to continue to educate multidisciplinary team members in spiritual care. Nonetheless, it is important that the role of chaplaincy is included in such education, so that those with specialist knowledge in spiritual care are called on to see the patients in greatest need (and requiring greatest expertise) wherever possible, particularly as in many countries there will never be enough chaplains to see all patients admitted to healthcare facilities. Generalist spiritual care is important for holistic care of patients, but it is also important that the boundary between generalist and specialist spiritual care is maintained. More research is needed into what determines those boundaries and how they should be implemented.

The opening up of new avenues for providing spiritual care that has occurred during COVID, such as telechaplaincy, is exciting and promises new opportunities for contact with patients and families who may previously have been difficult to access due to distance, visitor restrictions or other barriers. It is not surprising that this was introduced not just for COVID patients, but for all patients, given the hospital-wide measures required to minimise infection risk. The concurrent growth of community expertise in using e-communication that has occurred during the pandemic also increases the likelihood that such means of communication will continue to be acceptable to patients and families. It is interesting to note that some respondents considered these avenues even more effective than face-to-face communication, suggesting that research may be required to identify whether a particular modality is preferable for any particular chaplaincy encounter.

Moral injury is a predictable outcome when healthcare professionals are prevented from doing their job well (Bard & Bursztajn, 2020). Reports of suffering and undignified patient care witnessed while chaplains were limited in options to respond are distressing to read. This is of particular concern given the challenges many respondents experienced in trying to adequately engage in self-care. Within healthcare, moral distress has been repeatedly found to be correlated with burnout and intention to leave a workplace (Epstein et al., 2019) as well as taking a toll on the physical and mental health of healthcare professionals (Bard & Bursztajn, 2020; Sanderson et al., 2019). It will be important, once the pandemic has eased, that the fall-out from its impact is adequately addressed for all healthcare staff, including chaplains.

## Conclusion

There are inevitable challenges in developing methodologies for studies to be conducted in response to an emergency situation. This study is commendable to including the experiences of a large cohort across many countries. Qualitative methods are appropriate to explore a new topic, however the use of a survey rather than interviews of focus groups led by a trained researcher, while understandable, means that some themes may not have been fully investigated, and some meanings may have been misinterpreted. It is tempting to use leading questions when verbal prompts are not available. Nonetheless this is a valuable insight into the impact of the pandemic on chaplains internationally and gives much food for thought regarding future directions for the profession. Future studies should include more baseline data on the routine practices of chaplains so that changes to the norm can be quantified. It is sad that a pandemic was required to bring the importance of chaplaincy to the fore in so many places, and it now remains to extend that understanding more widely and ensure that it is not forgotten.
